# Advances and New Perspectives in Micro-Nanofabricated Sensors for Bioanalysis

**DOI:** 10.3390/bios14020087

**Published:** 2024-02-04

**Authors:** Amir Hatamie

**Affiliations:** 1 Department of Chemistry and Molecular Biology, University of Gothenburg, 41296 Gothenburg, Sweden; amir.hatami@gu.se; 2 Department of Chemistry, Institute for Advanced Studies in Basic Sciences (IASBS), Gava Zang, Zanjan 45137-66731, Iran

“Micro-Nanofabricated Sensors for Bioanalysis” represents a cutting-edge field in biosensing technology which leverages the integration of micro- and nanoscale fabrication techniques. These advancements aim to develop highly sensitive, miniaturized sensors tailored for biological analysis across a variety of samples. These sensors are specifically designed to detect and analyze biomolecules, such as proteins and neurotransmitters, and various biological samples, including single cells, brain tissue, and subcellular organelles, with high precision and sensitivity.

Current research in this field focuses on leveraging micro- and nanofabrication methods, such as photolithography, nanoimprinting, and microfluidics, to create sensor platforms with enhanced capabilities. These sensors commonly utilize various transduction mechanisms—using electrochemical, optical, or mechanical principles—to convert biological interactions into measurable signals. This enables the detection of biomarkers, enzymes, DNA sequences, proteins, and other analytes critical for medical diagnostics, environmental monitoring, and biotechnological applications.

In recent decades, integrating sensors with nanostructures has shown the potential to improve their capabilities and signal-to-noise ratio. Various nanostructures, including nanoparticles, nanotubes, nanowires, and nanocomposites, effectively enhance sensor performance and productivity. Developing new nanostructures promises the ability to further enhance sensor performance in the near future. The emergence of point-of-care devices and wearable biosensors is expected to revolutionize healthcare by enabling real-time monitoring and personalized clinical diagnostic applications.

Continuous innovation in fabrication techniques, material science, and signal processing, combined with interdisciplinary collaborations, will propel the evolution of micro-nanofabricated sensors. These advancements will lead to breakthroughs in bioanalysis and carry profound implications across various domains. Due to these significant advantages and their importance, this Special Issue is dedicated to collecting and presenting recent advances in a range of topics related to Micro-Nanofabricated Sensors for Bioanalysis.

